# Hydrogen Bond-Assisted Ultra-Stable and Fast Aqueous NH_4_^+^ Storage

**DOI:** 10.1007/s40820-021-00671-x

**Published:** 2021-06-10

**Authors:** Xikun Zhang, Maoting Xia, Haoxiang Yu, Junwei Zhang, Zhengwei Yang, Liyuan Zhang, Jie Shu

**Affiliations:** School of Materials Science and Chemical Engineering, Ningbo University, Ningbo, Zhejiang 315211 People’s Republic of China

**Keywords:** Aqueous ammonium ion batteries, Copper hexacyanoferrate, Ultra-long cycling performance, Excellent rate performance, Hydrogen bonds

## Abstract

Aqueous ammonium ion batteries are regarded as eco-friendly and sustainable energy storage systems. And applicable host for NH_4_^+^ in aqueous solution is always in the process of development. On the basis of density functional theory calculations, the excellent performance of NH_4_^+^ insertion in Prussian blue analogues (PBAs) is proposed, especially for copper hexacyanoferrate (CuHCF). In this work, we prove the outstanding cycling and rate performance of CuHCF via electrochemical analyses, delivering no capacity fading during ultra-long cycles of 3000 times and high capacity retention of 93.6% at 50 C. One of main contributions to superior performance from highly reversible redox reaction and structural change is verified during the ammoniation/de-ammoniation progresses. More importantly, we propose the NH_4_^+^ diffusion mechanism in CuHCF based on continuous formation and fracture of hydrogen bonds from a joint theoretical and experimental study, which is another essential reason for rapid charge transfer and superior NH_4_^+^ storage. Lastly, a full cell by coupling CuHCF cathode and polyaniline anode is constructed to explore the practical application of CuHCF. In brief, the outstanding aqueous NH_4_^+^ storage in cubic PBAs creates a blueprint for fast and sustainable energy storage.

**Supplementary Information:**

The online version contains supplementary material available at 10.1007/s40820-021-00671-x.

## Highlights

- Zero capacity fading after over 3000 cycles at 1 C.
- Only 6.4% capacity is lost when rate is increased by 50 times.
- Diffusion mechanism of formation and fracture of hydrogen bonds is proposed.

**Supplementary Information:**

The online version contains supplementary material available at 10.1007/s40820-021-00671-x.

## Graphical abstract

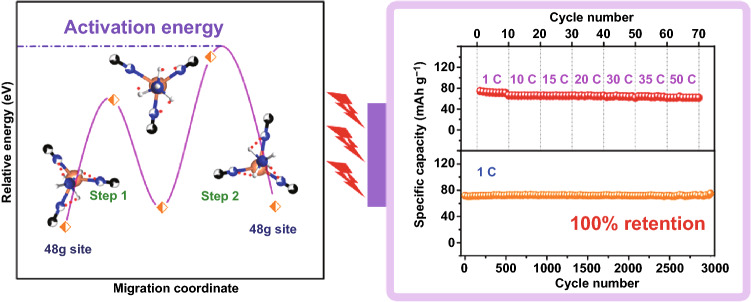

## Introduction

In 1990s, the first rechargeable aqueous lithium-ion battery was reported by Dahn to provide a substituent for organic batteries [1]. Obviously, the primary difference between previous studies is that aqueous solution served as electrolyte in their research rather than traditional organic electrolyte. During the past three decades, this pioneering work has inspired an increasing number of researchers to exploit more advanced rechargeable aqueous batteries [2–4]. Under this circumstance, aqueous monovalent and polyvalent ion batteries have been greatly developed and opened the path for practical applications. The inherent security, low price, and high ionic conductivity of aqueous batteries are irreplaceable by organic batteries [5–8]. Hence, the tremendous advances in aqueous batteries have opened a novel blueprint for the development of energy. To date, the research of aqueous batteries mainly focuses on the exploration of electrode materials and the optimization for practical performance.

According to the comparison between reported aqueous batteries, it is not difficult to find that present researches mainly focus on metallic carriers [9–13]. Nevertheless, proton (H^+^), hydronium (H_3_O^+^), and ammonium (NH_4_^+^) as inexpensive and sustainable nonmetallic carriers have rarely been studied [14–16]. In recent years, although some electrode materials that can be resided for H^+^ and H_3_O^+^, such as MoO_3_ and WO_3_ [17, 18], have been reported, their further applications are severely restricted due to the strong acidity of the electrolyte, which leads to strong corrosion of electrode materials and severe side reactions of hydrogen evolution [19–21]. In addition, as presented in Table S1, the infinitely abundant NH_4_^+^ not only exhibits moderate acidity, but also demonstrates smaller molecular weight (18 g mol^−1^) and hydrated ion radius (3.31 Å), which facilitates its rapid diffusion [22]. Therefore, aqueous ammonium ion batteries have been widely researched. For example, layered MXene materials and organic compounds are successfully exploited as excellent host for NH_4_^+^ storage [22–24]. Besides, the storage of NH_4_^+^ in transition metal sulfide is also realized by expanding the layer spacing of MoS_2_ and constructing the VS_2_/VO_x_ heterostructure [25, 26]. More importantly, V_2_O_5_ and MoO_3_ are reported as excellent host materials for fast NH_4_^+^ storage due to the formation of hydrogen bond between NH_4_^+^ and oxide [27, 28]. However, due to the limited performance or high price, these materials have not been able to achieve larger-scale practical applications. Thus, Prussian blue (PB) and its analogues (PBAs) are regarded as a potential host for novel NH_4_^+^ storage.

Cubic PBAs materials, which are described as A_x_L_y_[M(CN)_6_]z·nH_2_O, have long been popular with researchers for its unique rigid structure and ion transport channels [29–31]. The underlying reasons of the enthusiasm for PBAs may also come from the abundant species because L site can be displaced by numerous transition metals [32, 33]. For example, Lee et al. explored the Na_0.69_Fe_2_(CN)_6_ as cathode for magnesium ion battery and achieved the co-insertion of Mg^2+^ and Na^+^ at high voltage [34]. Besides, a host material for alkalis ion storage was synthesized when In atom resides in L site to form InFe(CN)_6_ [35]. In addition to the above materials that L site is replaced by single atom, the PBAs with different transition metals co-residing in L site are reported, such as K_1.85_Fe_0.33_Mn_0.67_[Fe(CN)_6_]_0.98_ and Na_2_Mn_0.15_Co_0.15_Ni_0.1_Fe_0.6_Fe(CN)_6_ [36, 37]. And PBAs materials are applied for aqueous NH_4_^+^ storage. For example, Ji et al. constructed the first “rocking chair” ammonium ion battery based on Ni-based PBAs and organic compound [24]. And the zero strain characteristic NH_4_^+^ of insertion in Berlin green is also explored [38]. Consequently, there is plenty space for researchers to exploit more promising PBAs as electrode materials.

In this research, we report cubic copper hexacyanoferrate (CuHCF) as host for aqueous NH_4_^+^ storage. Firstly, the favorable electrochemical performance is predicted by DFT calculations because of the formation of hydrogen bonds between H atoms in NH_4_^+^ species and N atoms in CuHCF. Secondly, the CuHCF shows outstanding electrochemical and kinetic performance as predicted in DFT calculations. For example, the specific capacity remains at about 77.5 mAh g^−1^ at 1 C even after 3000 cycles without any capacity loss. Besides, the rate performance demonstrates that only 6.4% of the specific capacity is lost when the current rate is increased by 50 times. And the capacity retention is as high as 72.5% after 30,000 cycles at 50 C. Then, a series of *ex situ* measurements are conducted to prove the reversible redox reaction and the existence of hydrogen bonds during the ammoniation/de-ammoniation progresses. Lastly, the NH_4_^+^ diffusion mechanism, which is based on continuous formation and fracture of hydrogen bonds, is proposed. And the practical application of CuHCF is proved by constructing a full cell. Therefore, this study not only provides a research method combining computation and experiment, but also explores the possibility of PBAs to realize fast and stable NH_4_^+^ storage.

## Results and Discussion

### Density Functional Theory Calculations

**Fig. 1:**
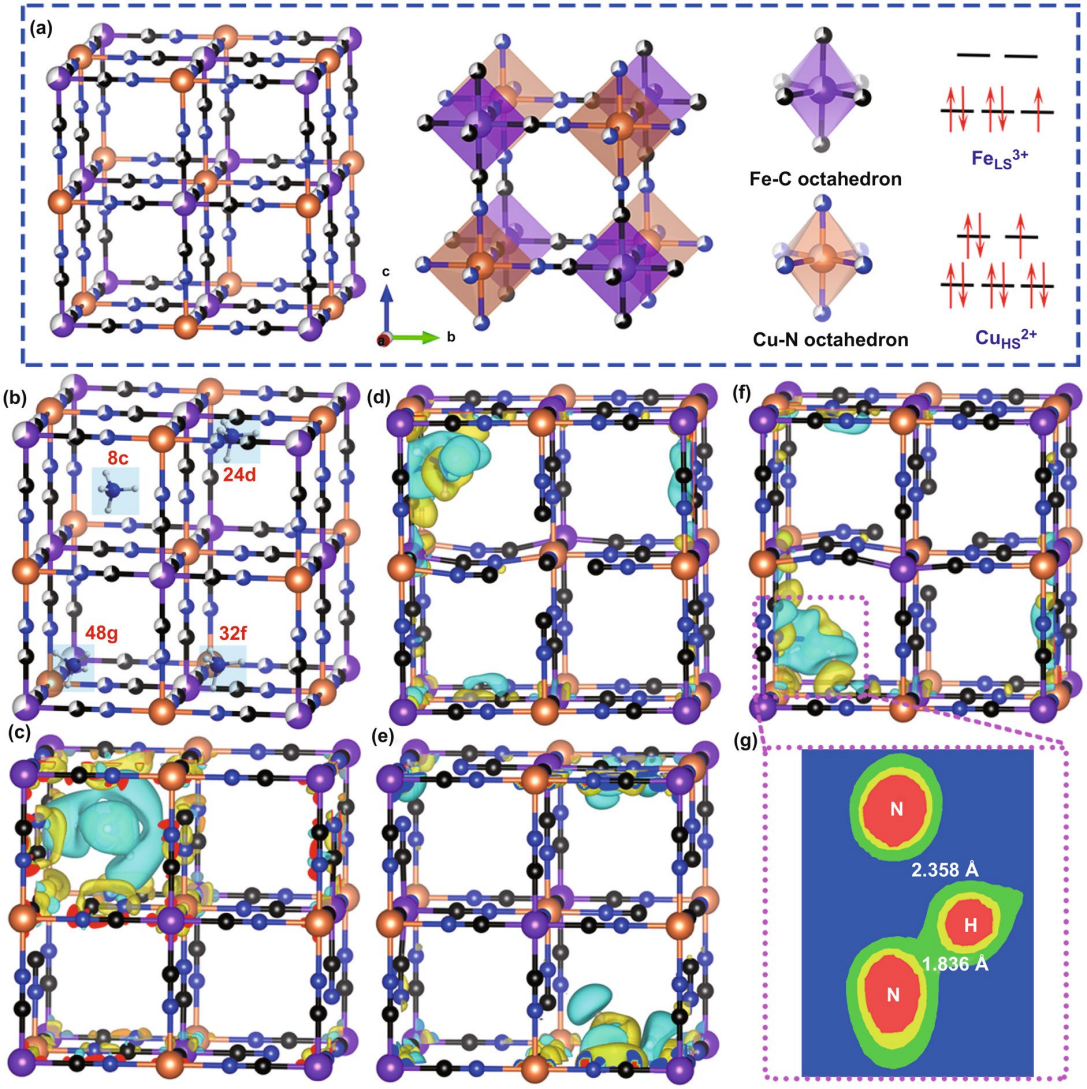
**a** Ideal crystal structure of cubic CuHCF and the schematic illustration of the electronic states for Fe and Cu atoms. **b** Schematic illustration of four possible interstitial positions for NH_4_^+^ storage in CuHCF. **c-f** Charge distribution when NH_4_^+^ locates at different sites from DFT calculations. **c** 8c site. **d** 24d site. **e** 32f site. **f** 48 g site. **g** Visualized hydrogen bonds and its length when NH_4_^+^ is inserted in 48 g site

Figure 1a presents the ideal crystal structure of CuHCF clearly, which reveals rigid cubic structure. The Fe, Cu, C, and N atoms are arranged orderly to form the skeleton, thus providing three-dimensional ion transport channels. Specifically, six C and N atoms are octahedral coordinated with central Fe and Cu atoms, respectively, where the two octahedrons are linked by –C≡N– bridges. And the C-coordinated Fe^3+^ belongs to low-spin state and shows one unpaired electron, which inclines to reduce to Fe^2+^ during the ion insertion. Then, density functional theory (DFT) calculations are performed to confirm the low energy model configurations of CuHCF when cation ions (such as NH_4_^+^) are inserted. As displayed in Figs. 1b and S1, there are four possible interstitial positions for NH_4_^+^ residence in cubic CuHCF, which can be described with Wyckoff notations as 8c, 24d, 32f, and 48 g. Besides, binding energy (*E*_b_) for different interstitial positions is calculated to estimate the relative stability of CuHCF when NH_4_^+^ is inserted. The calculated results show that NH_4_^+^ inclines to reside in 48 g site with the lowest *E*_b_ of -2.986 eV (Table S2), which originates from the formation of hydrogen bonds between the H atoms in NH_4_^+^ species and the N atoms in CuHCF to stabilize the system. Moreover, Fig. 1c–f demonstrates the charge distribution when NH_4_^+^ locates at different sites. Specifically, the length of hydrogen bond is about 1.836 Å at 48 g site and the charge is distributed along the hydrogen bonds (Fig. 1g), which facilitates rapid charge transfer. Therefore, the diffusion process based on hydrogen bonds between host and carrier may be beneficial to achieve superior kinetic performance.

### Physical Characterization of CuHCF

**Fig. 2:**
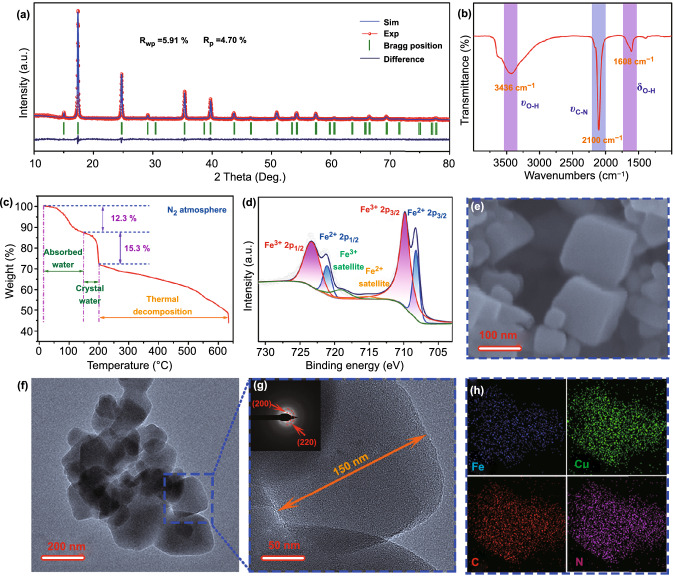
**a** Rietveld XRD pattern. **b** FTIR spectrum. **c** TGA curve from room temperature to 650 °C at a heating rate of 10 °C min^−1^ in N_2_ atmosphere. **d** XPS spectrum of Fe 2p region. **e** SEM image. **f-g** TEM images; the inset is the SAED image. **h** EDS mapping images

To confirm the inference in DFT calculations, the CuHCF is prepared by a direct co-precipitation method. Besides, the experimental powder X-ray diffraction (XRD) pattern and refined results, which were refined by Rietveld refinement method, are displayed in Fig. 2a. The XRD results confirm the high crystallinity and purity of CuHCF due to the matched and sharp diffraction peaks. In addition, according to the refined results (*R*_wp_ = 5.91%), the CuHCF delivers lattice unit cell volume of 1040.31 Å^3^ with *a* = *b* = *c* = 10.1326 Å and *α* = *β* = *γ* = 90°, which assigns to the space group of *Fm-3 m* (JCPDS No. 86-0514). Therefore, the broad ion transport channel provides potency for carrier residence. In order to further identify the physical ingredient in CuHCF, a series of measurements are performed. Firstly, Fourier transform infrared (FTIR) is deployed to analyze the coordination environment of −C≡N– ligands. As observed in Fig. 2b, a distinct stretching peak located at 2100 cm^−1^ that ascribes to –C≡N– is detected. Moreover, certain absorbed/crystal water is also verified due to the appearance of the stretching and bending peaks for O–H, which locates at 3437 and 1608 cm^−1^ [39–41]. Secondly, according to the above results, the content of absorbed/crystal water is quantified via thermogravimetric analysis (TGA) within 650 °C under N_2_ atmosphere (Fig. 2c). The weight loss in step one below 150 °C corresponds to 12.3% weight loss, which is attributed to the remove of absorbed water in CuHCF. Besides, the weight loss of 15.3% between 150 to 200 °C is attributed to the remove of crystal water [42, 43], corresponding to about 5.2 H_2_O per CuHCF unit.

Besides, X-ray photoelectron spectroscopy (XPS) is applied to illustrate the chemical composition and chemical valence of Fe and Cu elements in as-prepared CuHCF. As shown in Figs. 2d and S2, the sample exhibits mixed valence of Fe^3+^, Fe^2+^, Cu^2+^, and Cu^+^. Specifically, a pair of peaks located at 709.9 and 723.4 eV are bounded to the 2*p*_3/2_ and 2p_1/2_ spin-orbital of Fe^3+^, respectively. Likewise, the Fe^2+^ exhibits a couple of peaks at 708.2 and 721.1 eV [44, 45]. And similar phenomenon is detected in XPS spectra of Cu 2p. In fact, although Cu^+^ is not stable in air or aqueous solutions, it can be stable in the form of coordination compounds without being oxidized to Cu^2+^. Due to the fully occupied d orbital, there is no unpaired electron in the extra-nuclear of Cu^+^. And Cu^+^ coordinates with six N atoms in CuHCF, which reduces the electrostatic repulsion. Therefore, Cu^+^ can exist stably in CuHCF without being oxidized. Combined the above results with inductively coupled plasma optical emission spectrometry (ICP-OES) and elemental analysis, the exact formula of as-prepared CuHCF is Cu_2.95_[Fe(CN)_6_]_1.69_·5.2H_2_O, in which the ratios of Fe^3+^/Fe^2+^ and Cu^2+^/Cu^+^ are 3:1 and 7:1. And the detailed element contents are displayed in Table S3. The surface morphology of CuHCF is observed by scanning electron microscopy (SEM) and transmission electron microscopy (TEM). Figures 2e and S3 show the SEM images of CuHCF, which presents dispersive nanoparticles. And most particles exhibit regular cubic structure. The layout is similar to the random distribution of cubic boxes. Moreover, the TEM images in Figs. 2f and S4 further confirm the conclusion from SEM results. Clearly, the nanoparticles are uniformly dispersed in the field of vision and maintain their own cubic morphology. The edge length of CuHCF particle is about 150 nm as shown in Fig. 2g. In addition, the inset in Fig. 2g demonstrates the polycrystalline characteristics of cubic CuHCF and the (200) and (220) planes are clearly detected. Figure 2h indicates that the Fe, Cu, C, and N elements uniformly distribute in cubic nanoparticles of CuHCF. In addition, the specific surface is 381.5 m^2^ g^−1^ and the pore size is mainly distributed below 35 nm for CuHCF, which demonstrates a mesoporous structure (Fig. S5).

### Electrochemical Characterization of CuHCF

**Fig. 3:**
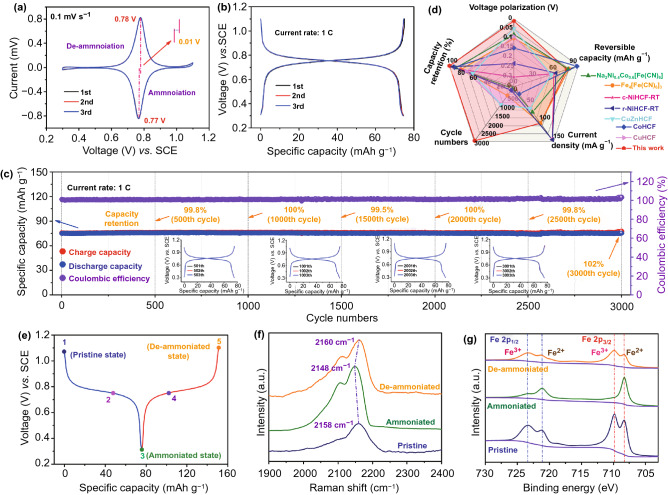
**a** First three CV curves at 0.1 mV s^−1^. **b** First three charge/discharge curves at current rate of 1 C. **c** Long-term cycling performance at 1 C; the insets are the GCD curves at different cycle numbers. **d** Comparison of cycling performance between the similar PBAs. **e** GCD curves at different charge/discharge states. **f** Ex situ Raman spectra of CuHCF electrode at pristine and ammoniated/de-ammoniated states. **g** Ex situ XPS spectra for Fe 2p at pristine and ammoniated/de-ammoniated states

**Fig. 4:**
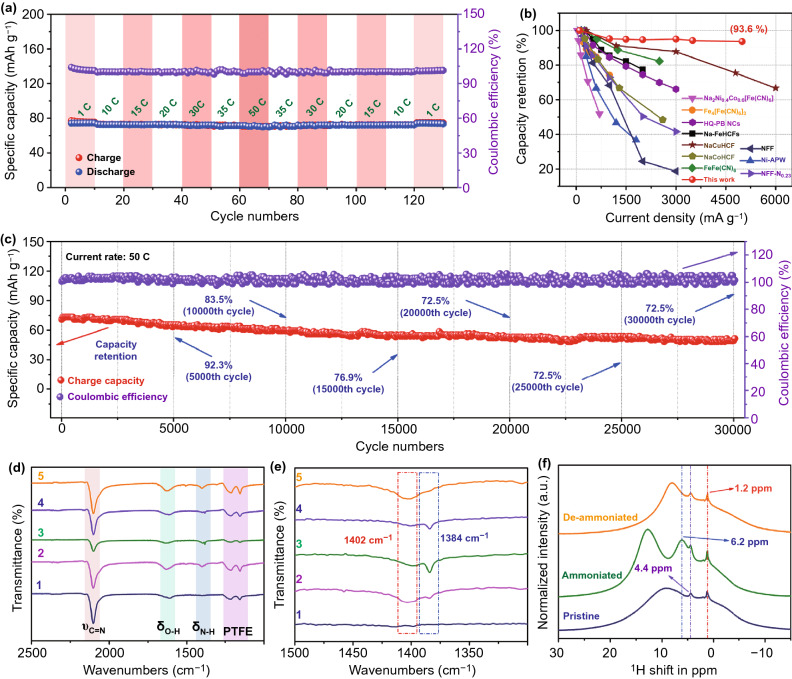
**a** Rate performance between 1 and 50 C. **b** Comparison of rate performance between this work and reported PBAs. **c** Long-term cycling performance at high current rate of 50 C. **d** Ex situ FTIR spectra of CuHCF electrode at pristine and ammoniated/de-ammoniated states. **e** Enlarged ex situ FTIR spectra between 1500 and 1300 cm^−1^. **f** Ex situ solid-state ^1^H NMR spectra at pristine and ammoniated/de-ammoniated states

The electrochemical performance of CuHCF is intensively demonstrated in Figs. 3 and 4, which is measured at a three-electrode battery in operating voltage range of 0.3–1.1 V. To remove the effect of trace K^+^ in the lattice of CuHCF, all the electrodes are pretreated prior to test. And the pretreatment process is displayed in Fig. S6. First of all, the cyclic voltammetry (CV) curves in Fig. 3a reveal that there is only one couple of reduction/oxidation peaks located at 0.77 and 0.78 V, which is ascribed to the ammoniation/de-ammoniation progresses of CuHCF during the cathodic and anodic scans, respectively. Besides, the reproducibility of CV curves and low voltage polarization of 0.01 V manifests the highly reversible electrochemical reaction of Fe^3+^/Fe^2+^ couple. Then, the galvanostatic charge/discharge (GCD) curves at a current rate of 1 C (1 C = 100 mA g^−1^) are shown in Fig. 3b. A couple of distinct slopes are clearly observed between 0.7–0.8 V, which is consistent with CV curves. And the first charge/discharge capacities are 75.1/74.9 mAh g^−1^, indicating an initial Coulombic efficiency of about 100%. Moreover, the complete coincidence of the first three GCD curves further indicates the reversibility of the ammoniation/de-ammoniation progresses in CuHCF and the high capacity retention rate. Figure 3c demonstrates the cycling performance of CuHCF at 1 C within ultra-long lifespan and the GCD curves at different cycles. Similar to the results in GCD tests, the first charge capacity and Coulombic efficiency are 75.8 mAh g^−1^ and 100%. Then, the capacity retention is 100% after 1000 cycles and the Coulombic efficiency also maintains at 100%, indicating a favorable cycling performance. More importantly, there is zero capacity fading after an ultra-long lifespan of 3000 cycles. The results show that cubic CuHCF can maintain its initial capacity and excellent Coulombic efficiency upon repeated cycles, which shows the structural stability and the possibility of practical application for CuHCF. And the cycling performance is far superior to other PBAs as displayed in Fig. 3d [46–48].

In order to explore the nature of excellent cycling performance, ex situ Raman and XPS measurements are conducted. Considering the effect of chemical environment on –C≡N– ligands, ex situ Raman spectroscopy is deployed to evaluate the average valence state of Fe element in CuHCF during ammoniation/de-ammoniation progresses. And ex situ Raman spectra are recorded at different states of charge (Fig. 3e) as shown in Fig. 3f. A distinct peak located at 2158 cm^−1^ is observed in pristine CuHCF, which is ascribed to Fe^3+^–C≡N– groups [41]. After discharge progress (ammoniated state), a visible red shift, which moves to 2148 from 2158 cm^−1^, is detected. And this phenomenon is ascribed to the low average valence state of Fe element in CuHCF, indicating the reduction progress of Fe^3+^ during NH_4_^+^ insertion [49]. Besides, opposite change occurs in de-ammoniation progress and the peak turns back to 2160 from 2148 cm^−1^, manifesting the oxidation of Fe^2+^ to Fe^3+^. Consequently, this variation reflects the high reversibility of Fe^3+^/Fe^2+^ couple. Furthermore, ex situ XPS spectroscopy is applied to verify the content and variation of Fe^2+^ and Fe^3+^ in CuHCF. As depicted in Fig. 3g, compared with pristine sample, most Fe^3+^ are reduced to Fe^2+^ after NH_4_^+^ insertion in CuHCF lattice (ammoniated state). In addition, upon NH_4_^+^ extraction from CuHCF, Fe^2+^ is oxidized to Fe^3+^ and the distribution of Fe^3+^/Fe^2+^ content is similar with the pristine sample, which confirms the reversible redox reaction of Fe^3+^/Fe^2+^ couple. And the XPS results are consistent with the conclusions in Raman spectra. In contrast, the valence state of Cu rarely changes during ammoniation/de-ammoniation progress as depicted in Fig. S7. The highly reversible redox reduction of Fe^3+^/Fe^2+^ couple observed in both ex situ Raman and XPS results manifests the low voltage polarization and excellent cycling performance.

Generally speaking, the cycling performance at low current density is usually the basis of rate performance. For another, the fast charge transfer originated from the formation of hydrogen bonds is also a favorable factor for high rate performance. Therefore, the rate performance is surveyed to prove the results in DFT calculations. As depicted in Figs. 4a and S8, CuHCF delivers a charge capacity of about 76 mAh g^−1^ at 1 C. Then, when the current rate is increased to 35 C, the capacity drops to 72 mAh g^−1^, indicating a high capacity retention of 94.7% compared with 1 C. Even at a higher rate of 50 C, the capacity maintains at 71 mAh g^−1^ and the capacity retention is as high as 93.6%. Besides, the capacity increases to 76 mAh g^−1^ when the current rate decreases to 1 C. And the Coulombic efficiency remains at 100% throughout the change in current rates. Therefore, the rate performance demonstrates that a 50-fold increase in current rate only results in a 6.4% total capacity loss, which is an outstanding advantage over other electrode materials in Fig. 4b and Table S4 [46–48, 50–54]. Furthermore, the long-span cycling performance at a high current rate of 30 C is shown in Figs. S9 and S10. High capacity retention of 74.5% is achieved after 23,000 cycles. Even at higher current rate of 50 C, the capacity retention is as high as 72.5% after over 30,000 cycles (Figs. 4c and S11), corresponding a low capacity decay of 0.001% per cycle. Therefore, the outstanding rate performance is the experimental evidence of rapid charge transfer.

Then, to illustrate the favorable high rate performance, pseudocapacitance and diffusion contributions are surveyed by investigating the kinetic feature of CuHCF at various scan rates of 0.1–2.0 mV s^−1^ [55, 56]. As presented in Fig. S12, at 2.0 mV s^−1^, the main ratio (blue area) accounts for 87% of the total capacity, which is attributed to the capacitive contribution. Furthermore, the capacitive contributions at other scan rates demonstrate that the capacitive contributions ascend gradually with the increase in scan rates [57–59]. Specifically, the capacitive contributions are 68%, 72%, 75%, 79%, 82%, and 87% at 0.1, 0.2, 0.3, 0.5, 1.0, and 2.0 mV s^−1^, respectively. The dominated pseudocapacitive contribution reflects the non-diffusion behavior and mainly stems from the topo-chemistry reaction mechanism between NH_4_^+^ and cubic CuHCF. Consequently, the high capacitive contribution is the reasonable explanation of high rate performance for CuHCF nanoparticle. Besides, the transfer resistance and diffusion coefficient for NH_4_^+^ transport are evaluated by electrochemical impedance spectra (EIS). As displayed in Fig. S13, the transfer resistances are 1.5 and 2.0 Ω for ammoniated and deammoniated CuHCF, respectively. In addition, the corresponding calculated diffusion coefficients are 1.20 × 10^–11^ and 4.53 × 10^–12^ cm^2^ s^−1^.

After unveiling the reversible redox reduction of Fe^3+^/Fe^2+^ couple in Fig. 3, the inner nature of rapid charge transfer is also explored. It is well known that the hydrogen bonds are regarded as particular chemical bonds between H and N or O atoms. Therefore, ex situ FTIR and solid-state nuclear magnetic resonance (SSNMR) are conducted to detect the changes in chemical environment and the species of protons in CuHCF. As shown in Fig. 4d, compared with the pristine sample, all the stretching peaks of –C≡N– bonds maintain at about 2100 cm^−1^, indicating the stable basic framework of CuHCF during the ammoniation/de-ammoniation processes. Besides, in addition to the unvaried –C≡N– bonds, the bending peaks of N–H located at about 1400 cm^−1^ are also detected and the enlarged region is displayed in Fig. 4e. It is clear that the bending peaks of N–H are composed of two peaks, which locate at 1402 and 1384 cm^−1^, respectively. The former is ascribed to the non-bonded H atoms in NH_4_^+^ species. And the latter can be ascribed to the hydrogen bonds, which are formed between the H atoms in NH_4_^+^ and N atoms in CuHCF. Specifically, its intensity gradually increases from pristine sate to ammoniated state and then decreases during de-ammoniation process. Therefore, the intensity evolution manifests the alternant formation and fracture of hydrogen bonds during ammoniation/de-ammoniation processes.

To further confirm the existence and evolution of hydrogen bonds, ex situ SSNMR is conducted to verify the species of protons at different states of charge and the ^1^H NMR spectra are shown in Fig. 4f. Firstly, in the pristine sample, the main resonances at 4.4 and 9.0 ppm are assigned to the hydrogen bonds of adsorbed/lattice water [60, 61], respectively. And the resonating peak at 1.2 ppm may be ascribed to the C-H bonds, which is originated from the impurity in acetylene black or binder. Then, a new resonance located at 6.2 ppm is detected at ammoniated state, which is attributed to the hydrogen bonds [62, 63], indicating the insertion of NH_4_^+^ in CuHCF and formation of hydrogen bonds between the H atoms in NH_4_^+^ and N atoms in CuHCF. And this phenomenon is completely consistent with the ex situ FTIR results. Besides, the formation of hydrogen bonds rarely affects the resonances at 1.2 and 4.4 ppm, but causes the downfield shift of the resonating peak at 9.0 ppm, which moves to about 12.0 ppm. Lastly, the resonating peak of hydrogen bonds disappears after the de-ammoniation process, indicating NH_4_^+^ extraction from CuHCF. Then, the resonance at 12.0 ppm returns to 9.0 ppm, which is the same as the pristine sample. And the changes in protonic species indicate the reversible ammoniation/de-ammoniation processes in CuHCF lattice. Therefore, the diffusion process based on hydrogen bond is beneficial to achieve excellent kinetic performance of NH_4_^+^ storage in cubic CuHCF.

After the above analysis, it can be found that the performance of CuHCF is closely related to its structure and properties. Firstly, CuHCF demonstrates rigid cubic structure and large ion transport channels for NH_4_^+^ transport. And the structure is considerable stable in aqueous electrolyte after repeated ammoniation/de-ammoniation progresses. Secondly, the high reversible redox reaction of Fe^3+^/Fe^2+^ couple is the inherent essence of ultra-stable long-term cycling performance. And the ex situ XPS and Raman measurements confirm the high reversibility. Lastly, the excellent cycling performance provides a favorable basis for the rate performance. Besides, the high pseudocapacitive contribution and diffusion coefficient promote the fast NH_4_^+^ transport in CuHCF lattice. More importantly, the hydrogen bond between NH_4_^+^ and CuHCF, which is detected by FT-IR and NMR measurements, facilitates rapid charge transfer. Thus, CuHCF also demonstrates outstanding rate performance.

### Exploration on Reaction Mechanism

**Fig. 5:**
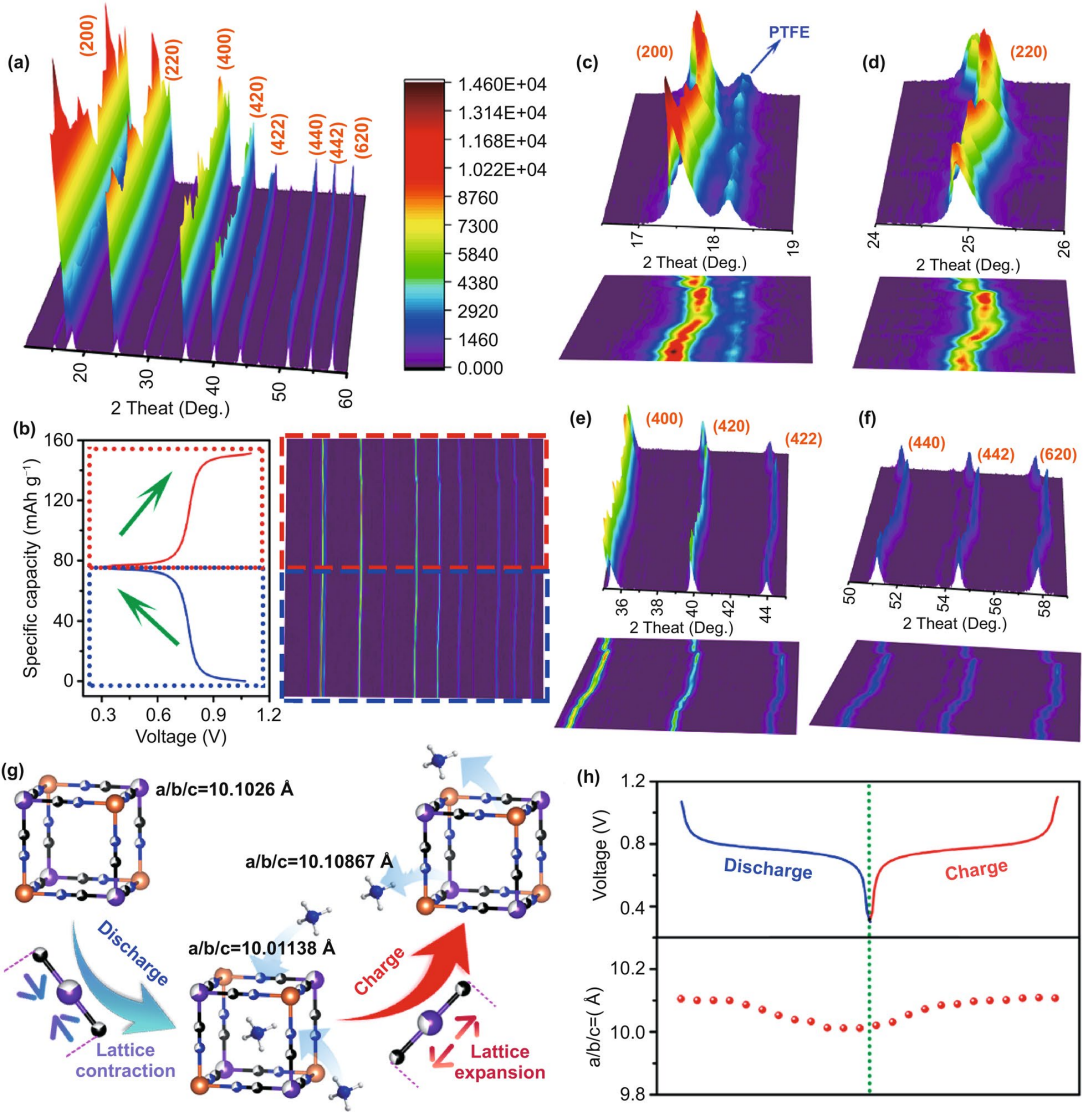
**a** Overall XRD patterns and the two-dimensional color map. **b** Corresponding GCD curves and projection view of XRD patterns. **c–f** Enlarged regions of 16°–19°, 23°–27°, 35°–45°, and 50°–60°. **g** Schematic illustration of the changes in Fe–C bond distance during ammoniation/de-ammoniation progresses. **h** Lattice parameter changes during charge/discharge cycle

To probe the structural evolution of CuHCF during ammoniation/de-ammoniation progresses, the XRD patterns are monitored at different states of charge within the first cycle. The overall XRD spectra and corresponding charge/discharge curves are presented in Fig. 5a, b. Generally speaking, the CuHCF maintains the same cubic structure with pristine samples after the first cycle because no impure phase is detected in all XRD spectra, which indicates as a solid solution reaction. Moreover, the enlarged figures of different crystal planes are displayed in Fig. 5c, f. All the crystal planes demonstrate the same evolution trend during repeated charge/discharge progresses. Specifically, the diffraction peaks move to the high angles during ammoniation progress (discharge) and turn back to the original position during de-ammoniation progress (charge), which is corresponding to the lattice contraction and expansion as schematically shown in Fig. 5g. And this evolution is contrary to the materials previously reported [64]. Here, the lattice contraction is derived from the decrease in Fe–C bond distance during reduction progress. Specifically, during the discharge process, NH_4_^+^ insertion takes place in the lattice of CuHCF, which results in the reduction of Fe^3+^ to Fe^2+^, thus leading to the decrease in Fe–C bond distance and the lattice contraction of CuHCF. Then, the reverse phenomenon occurs during charge process, resulting in the lattice expansion. According to the refined XRD result, NH_4_^+^ resides in 48 g site in cubic CuHCF. Figure 5h exhibits the changes of lattice parameters (a/b/c), which decreases to 10.01138 Å (ammoniation) and then increases to 10.10867 Å (de-ammoniation). The slight change in lattice parameters is related to the stable framework and thus resulting in favorable cycling and rate performance. In addition, after 3000 cycles, CuHCF still maintains its initial cubic structure, indicating its structural stability (Fig. S14).

**Fig. 6:**
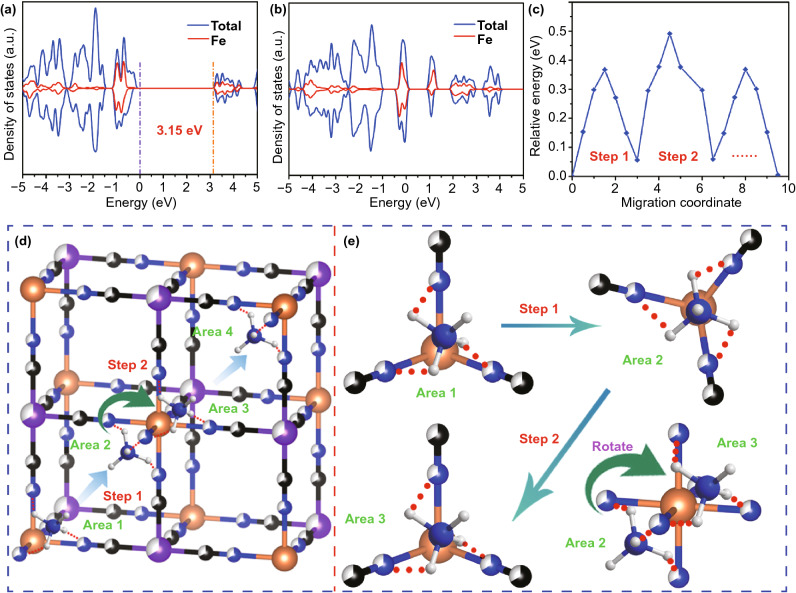
**a-b** Density of states for Fe atom in pristine and ammoniated CuHCF. **c** Changes of diffusion activation energy during ammoniation/de-ammoniation progresses. **d** Schematic illustration of NH_4_^+^ diffusion from 48 g site to another. **e** Detailed view of NH_4_^+^ diffusion

The study on diffusion mechanism is a deeper understanding than the study of structural and component changes. It is well known that the configuration of extra-nuclear electron of Fe^3+^ is [Ar]3d^5^. Therefore, there is only one unpaired electron in the low-spin Fe^3+^ in CuHCF. After cation insertion, Fe^3+^ is reduced to Fe^2+^, and the unpaired electron is zero. However, the configuration of extra-nuclear electron of Cu^2+^ is [Ar]3d^9^, which shows only one unpaired electron in both high-spin and low-spin states. As shown in Fig. 6a, b, in the pristine sample, CuHCF shows a small band gap of about 3.15 eV. At ammoniated state, the changes in electronic density of states are mainly concentrated in Fe atoms, while Cu atoms are almost unchanged (Fig. S15), which is completely consistent with XPS results. And some unoccupied spin states above Fermi level are noticed, which may be caused by changes in the valence state of Fe atoms. Besides, the diffusion process of NH_4_^+^ in CuHCF and corresponding diffusion activation energy are displayed in Fig. 6c–e. When NH_4_^+^ is inserted in 48 g site, the H atoms form hydrogen bonds with the N atoms in CuHCF. Therefore, the diffusion process of NH_4_^+^ from one 48 g site to another is mainly based on the continuous formation and fracture of hydrogen bonds. At the beginning of diffusion process, the total energy of the system is increased, which results in the fracture of hydrogen bonds when the activation energy reaches its maximum value (~ 0.37 eV). Then, NH_4_^+^ spreads forward until new hydrogen bonds are formed and energy is released, thus leading to the decrease in total energy (step 1). And step 2 is similar to step 1, but there are differences. Firstly, the fracture process of hydrogen bond is the same as step 1. Secondly, NH_4_^+^ is rotated when the hydrogen bonds are fractured completely, and the activation energy of the system increases continuously in this process (~ 0.49 eV). Therefore, the total energy is higher than that in step 1. Lastly, the new hydrogen bonds are formed in next 48 g site and the total energy is decreased, which is the same as step 1. And the visual NH_4_^+^ diffusion progress based on the continuous formation and fracture of hydrogen bond is displayed in Video S1.

### Full Cell Application

**Fig. 7:**
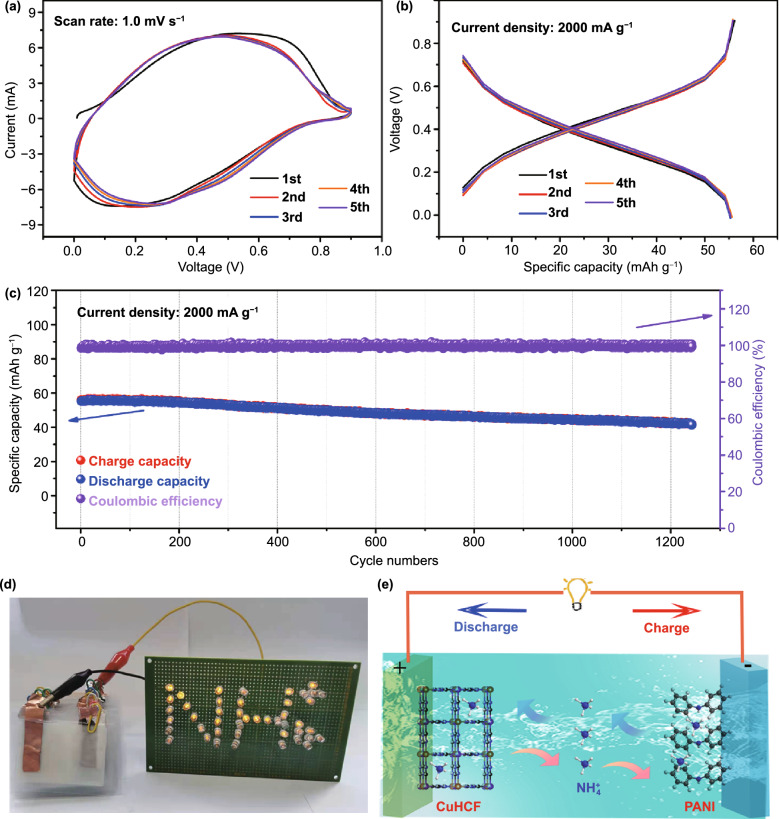
**a** Initial five CV curves of CuHCF//PANI full cell. **b** Initial five GCD curves at current density of 2000 mA g^−1^. **c** Cycling performance at 2000 mA g^−1^. **d** An LEDs array powered by CuHCF//PANI full cell. **e** Schematic illustration of CuHCF//PANI full cell

The practical application of CuHCF is further exploited by coupling pre-ammoniated CuHCF cathode with polyaniline (PANI) anode to fabricate CuHCF//PANI full cell. And the first five CV curves at voltage window of 0.0–0.9 V are displayed in Fig. 7a. Besides, the corresponding GCD curves at current density of 2000 mA g^−1^ are shown in Fig. 7b, which demonstrates charge/discharge capacities of 56.1/55.3 mAh g^−1^ and high initial Coulombic efficiency of 95.1%. More importantly, the charge capacity of the CuHCF//PANI full cell drops to 41.7 from 56.1 mAh g^−1^ after over 1240 cycles, indicating a high capacity retention of 74.3% (Fig. 7c). Figure 7d exhibits the light-emitting diodes array with “NH_4_^+^” shape powered by CuHCF//PANI full cell and further proves their possibility of practical application. The operation mechanism of CuHCF//PANI full cell is visually depicted in Fig. 7e, which is based on the “rocking-chair” insertion/extraction of NH_4_^+^ between CuHCF cathode and PANI anode. Specifically, during the charge process, NH_4_^+^ extracts from pre-ammoniated CuHCF cathode into electrolyte, and the oxidation reaction of Fe^2+^ to Fe^3+^ occurs. Meanwhile, NH_4_^+^ inserts into the PANI anode from electrolyte and electrons are transferred through an external circuit. Then, NH_4_^+^ extracts from PANI and inserts into CuHCF simultaneously during the discharge process, thus constituting the “rocking-chair” operating mechanism of NH_4_^+^, which guarantees the continuous operation of CuHCF//PANI full cell. Therefore, the possibility of practical application is verified.

## Conclusions

In conclusion, CuHCF demonstrates outstanding performance for aqueous NH_4_^+^ storage as predicted in DFT calculations. On the one hand, CuHCF shows small voltage polarization about 0.01 V and ultra-long cycling performance with zero capacity fading after over 3000 cycles, which manifests the reversible redox reaction of Fe^3+^/Fe^2+^ couple in CuHCF. And the result is proved by ex situ Raman and XPS measurements. On the other hand, the rate performance demonstrates that the capacity decreases by only 6.4% when the current rate is increased by 50 times. Besides, after over 30,000 cycles, the capacity retention is as high as 72.5% at 50 C, corresponding to a low capacity decay of 0.001% per cycle. The favorable rate performance is mainly originated from the formation of hydrogen bonds and then resulting in fast charge transport, which can be observed in ex situ FTIR and solid-state ^1^H NMR results. In addition, kinetic property and research on structural evolution further verify the high pseudocapacitance contributions and stable cubic structure of CuHCF. Lastly, the diffusion mechanism of “continuous formation and fracture of hydrogen bonds” is presented. Hence, the cubic CuHCF may provide an infinite development space for stable and fast aqueous NH_4_^+^ storage.

## Supplementary Information

Below is the link to the electronic supplementary material.

Supplementary file1 (PDF 2689 KB)

Supplementary file2 (MP4 8517 KB)
