# Supplementary material for: Hydrogen Bond-Assisted Ultra-Stable and Fast Aqueous NH4+ Storage
Source: Nanomicro Lett. 2021 Jun 10;13:139. doi: 10.1007/s40820-021-00671-x (PMC8192672; doi:10.1007/s40820-021-00671-x)
Supplement: Supplementary file 1 — Supplementary file1 (PDF 2689 KB) [file 40820_2021_671_MOESM1_ESM.pdf]

Supporting Information for

Hydrogen Bond Assisted Ultra-Stable and Fast Aqueous NH_4^+ Storage

Xikun Zhang[1], Maoting Xia[1], Haoxiang Yu[1], Junwei Zhang[1], Zhengwei Yang[1], Liyuan Zhang[1], Jie Shu[1, *]

[1]School of Materials Science and Chemical Engineering, Ningbo University, Ningbo, Zhejiang, 315211, P. R. China

*Corresponding author. E-mail: shujie@nbu.edu.cn (Jie Shu)

S1 Experimental Section

S1.1 Material Synthesis

In this research, $Cu_3[Fe(CN)_6]_2$ (CuHCF) was prepared via a simple co-precipitation method with $K_3[Fe(CN)_6]$ and anhydrous $CuSO_4$ as start precursors. Firstly, 1.6471 g $K_3[Fe(CN)_6]$ was dissolved in 50 mL deionized (DI) water to form solution A. Then, 1.5969 g anhydrous $CuSO_4$ was added in 50 mL DI water to form solution B. Lastly, under magnetometric stirring, solution A and solution B were added dropwise simultaneously to 100 mL DI water. The green precipitates were collected by centrifugation, washed with deionized water and ethanol, and then dried in a vacuum oven at 60 °C.

S1.2 Material Characterization

The Bruker AXS D8 Advance diffractometer (Cu-Kα radiation; receiving slit, 0.2mm; scintillation counter, 40 mA; 40 kV) was applied to collect the powder X-ray diffraction (XRD) patterns. The morphology and structure of CuHCF were observed by scanning electron microscope (SEM, Sirion 2000, FEI) and transmission electron microscope (TEM, JEM-2100 HR). Brunauer-Emmett-Teller surface areas were measured using Tristar II 3020 instrument by nitrogen adsorption at 77 K. Fourier transform infrared (FTIR) spectra were carried out by a 60-SXB IR spectrometer between 500 and 4000 cm^{-1}. X-ray photoelectron spectra (XPS) were measured on a VG MultiLab 2000 system with a monochromatic Al-Kα X-ray source and all binding energies were referenced to the C 1s peak of 284.8 eV. The chemical compositions of CuHCF was confirmed by inductively coupled plasma optical emission spectrometry (ICP-OES, Perkin Elmer 2100 DV) on Cu and Fe elements, elemental analysis (Elementar Vario Macro Cube) on C and N elements, and thermogravimetric analysis (TGA, DTG-60H, Shimadzu, Japan) on H_2O. Raman analyses were conducted in the range 200-3200 cm^{-1} with resolution 4 cm^{-1} using a Ranishaw InVia spectrometer equipped with a 532 nm green laser operated at a power of 0.5 mW. The solid-state nuclear magnetic resonance (SSNMR) spectra were measured by JNM-ECZ600R at room temperature, which was equipped with a Chemagnetics-Varian 3.2 mm pencil MAS probe and the magic-angle

spinning (MAS) rotational frequency was 15 kHz and the pulse delay was 4 s.

S1.3 Electrochemical Measurements

The electrochemical performance of CuHCF was examined in a three-electrode cell, which is composed of working electrode (active materials), counter electrode (Pt sheet), reference electrode (saturated calomel electrode (SCE), E = 0.244 V *vs.* standard hydrogen electrode), and aqueous electrolyte (2.0 M NH_4NO_3). In addition, the working electrodes were prepared by mixing active materials, acetylene black, and polytetrafluoroethylene binder at a weight ratio of 8:1:1. The slurry was cast on carbon paper current collector and dried at 70 °C in vacuum oven. And the active mass loading of each electrodes was about 5.0 mg cm^{-2}. The free-standing films are applied to perform ex-situ Raman, XPS, FTIR, and SSNMR tests. Galvanostatic charge/discharge (GCD) tests were examined using a multichannel LANHE CT2001A battery testing instrument at room temperature. Cyclic voltammetry (CV) was tested by a Bio-Logic VSP electrochemical workstation from 0.3 to 1.1 V. And electrochemical impedance spectroscopy (EIS) was also carried out on the Bio-Logic VSP workstation. The full cell was fabricated with pre-ammoniated CuHCF cathode and polyaniline (PANI) anode at a mass ratio of 1:1. And the active mass loading of each electrode was about 5.0 mg cm^{-2}.

S1.4 Computational Details

We employed the Vienna ab initio package (VASP) [S1, S2] to perform all spin-polarization density functional theory (DFT) calculations within the generalized gradient approximation (GGA) using the Perdew-Burke-Ernzerhof (PBE) [S3] formulation. We chose the projected augmented wave (PAW) potentials [S4, S5] to describe the ionic cores and take valence electrons into account using a plane wave basis set with a kinetic energy cutoff of 400 eV. Partial occupancies of the Kohn-Sham orbitals were allowed using the Gaussian smearing method and a width of 0.05 eV. The electronic energy was considered self-consistent when the energy change was smaller than 10^{-6} eV. A geometry optimization was considered convergent when the energy change was smaller than 0.05 eV $Å^{-1}$. In addition, for the Fe atoms, the U schemes need to be applied, and the U has been set as 3.2 eV. Finally, the binding energies (E_{ads}) were calculated as $E_{ads} = E_{ad/sub} - E_{ad} - E_{sub}$, where $E_{ad/sub}$, E_{ad}, and E_{sub} are the total energies of the optimized adsorbate/substrate system, the adsorbate in the gas phase, and the clean substrate, respectively. Besides, NH_4^+ ions migration barrier energies had been evaluated using the climbing nudged elastic band (CI-NEB) methods.

S2 Supplementary Tables and Figures

Table S1 Comparison of physical parameters between common monovalent and polyvalent ions

Ions	Molar mass (g mol^{-1})	Ionic radius (Å)	Hydrated radius (Å)	Acidity (pKa)	Crustal abundance (mass%)
Li^+	6.9	0.94	3.82	13.8	17 ppm
Na^+	23.0	1.17	3.58	14.2	2.3
K^+	39.1	1.49	3.31	14.5	1.5
Mg^{2+}	24.3	0.72	4.28	11.4	2.9
Zn^{2+}	65.4	0.74	4.30	9.6	79 ppm
Ca^{2+}	40.1	1.00	4.12	12.7	5.0
Al^{3+}	27.0	0.53	4.80	4.7	8.2
H_3O^+	19.0	1.15	2.80	-1.74	--
NH_4^+	18.0	1.48	3.31	9.2	--

Table S2 Binding energies for $NH_4Cu_3[Fe(CN)_6]_2$ at different sites

Sites	24d site	48g site	8c site	32f site
Binding energy (eV)	-2.973	-2.986	-1.251	-0.398

Table S3 Chemical compositions of CuHCF from ICP-OES analysis, elemental analysis, and TGA

Component	Cu	Fe	C	N	O
wt.%	29.3	14.8	19.0	22.2	13.2

Table S4 Comparison of electrochemical performance between CuHCF and similar PBAs

Materials	Electrolyte	Working Window	Cycling Performance[a)]	Rate Performance[b)]	Refs.
$K_2CoFe(CN)_6$	22 M CF_3SO_3K	0-1.2 V	70%/1000/0.6	100/90/77/74/66 at 0.02/0.04/0.06/0.08/0.1	[47]
$KNaNi[Fe(CN)_6]$	0.6 M NaCl	0-0.85 V	83%/200/0.145	58/50/45/40 at 0.145/0.29/0.58/1.16	[48]
$Na_{1.2}Ni[Fe(CN)_6]$	0.6 M NaCl	0-0.85 V	95%/200/0.145	63/60/53/50 at 0.145/0.29/0.58/1.16	[48]
$(CuZn)_x[Fe(CN)_6]_2$	0.1 M $ZnSO_4$	0.2-1.1 V	64%/1000/0.06	--	[49]
$FeFe(CN)_6$	1 M Na_2SO_4	-0.2-1.1 V	83%/500/1.25	122/120/110/91 at 0.25/0.625/1.25/2.5	[51]
$NaNiFe[Fe(CN)_6]$	1 M $NaNO_3$	-0.2-1.2 V	73%/1000/1	105/101/100/91/80/58/49 at 0.1/0.2/0.3/0.5/1/2/3	[52]
$Fe_4[Fe(CN)_6]_3$	1 M Na_2SO_4	-0.05-1.3 V	96%/200/1.25	126/115/107/100/94/88/83 at 0.25/0.5/1/1.5/2/2.5/3	[53]
$Na_2CuFe(CN)_6$	1 M Na_2SO_4	0-1.8 V	93%/500/0.3	56/52/49/43/38 at 0.3/1.2/3/4.8/6	[54]
$Na_2CoFe(CN)_6$	1 M Na_2SO_4	0-1.0 V	90%/800/0.13	125/120/92/84/61 at 0.13/0.26/0.65/1.3/2.6	[55]
$Cu_3[Fe(CN)_6]_2$	2 M NH_4NO_3	0.3-1.1 V	102%/3000/0.1	76/72/72/72/72/72/71 at 0.1/1/1.5/2/3/3.5/5	This work

[a)] The cycling performance is summarized as capacity retention/cycle numbers/current density (A g^{-1}).

[b)] The rate performance is summarized as capacity (mAh g^{-1}) at certain current density (A g^{-1})

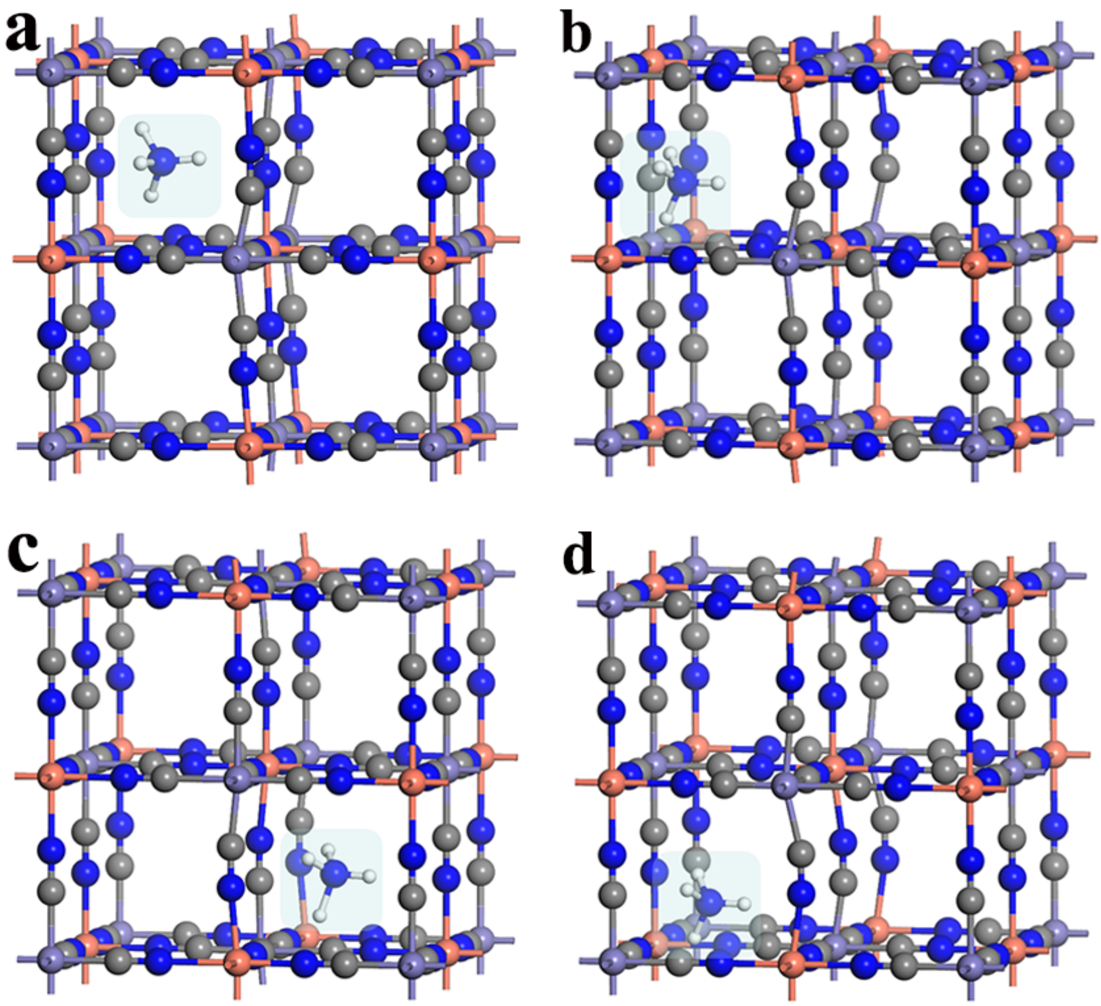

Fig. S1 Possible intercalation positions for $Cu_3[Fe(CN)_6]_2$. **a** 8c site. **b** 24d site. **c** 32f site. **d** 48g site

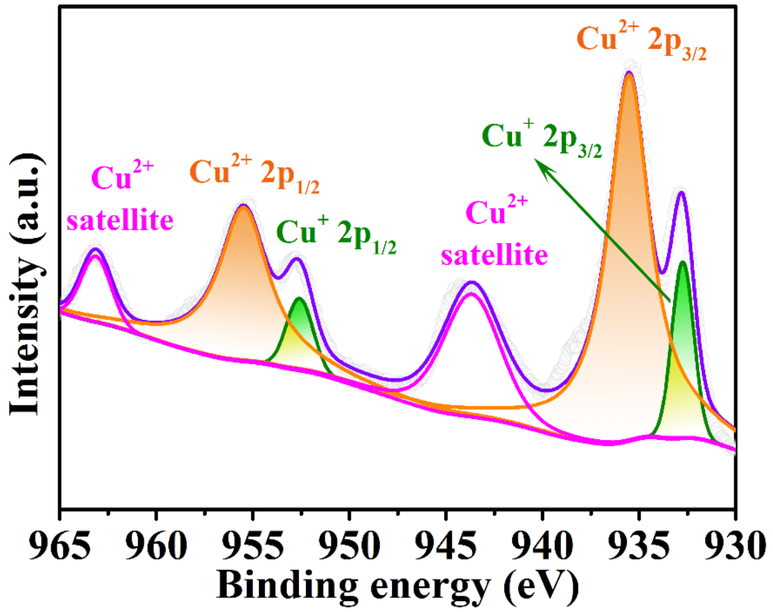

Fig. S2 XPS spectrum of Cu 2p region

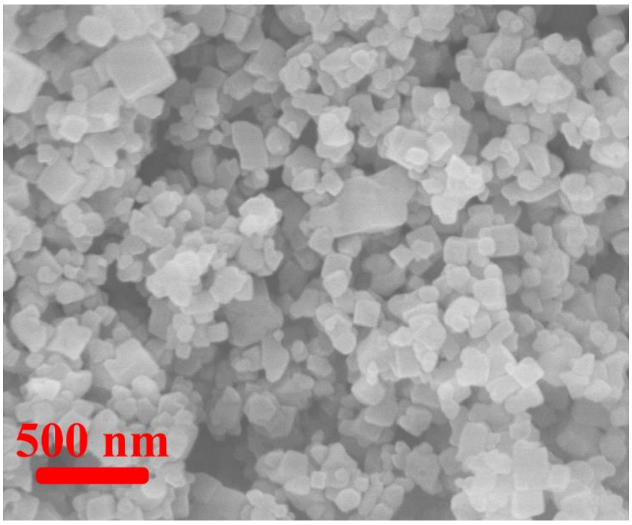

Fig. S3 SEM image of CuHCF

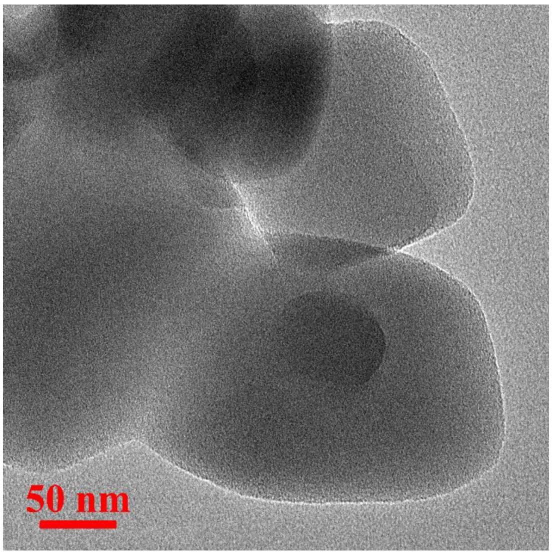

Fig. S4 TEM image of CuHCF

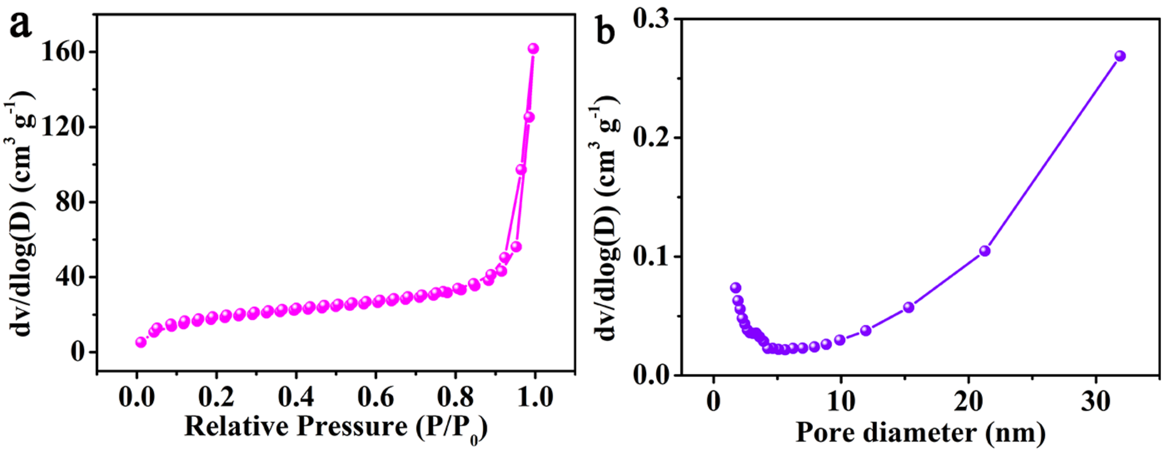

Fig. S5 **a** Nitrogen adsorption-desorption isotherm of CuHCF. **b** The corresponding pore size distribution

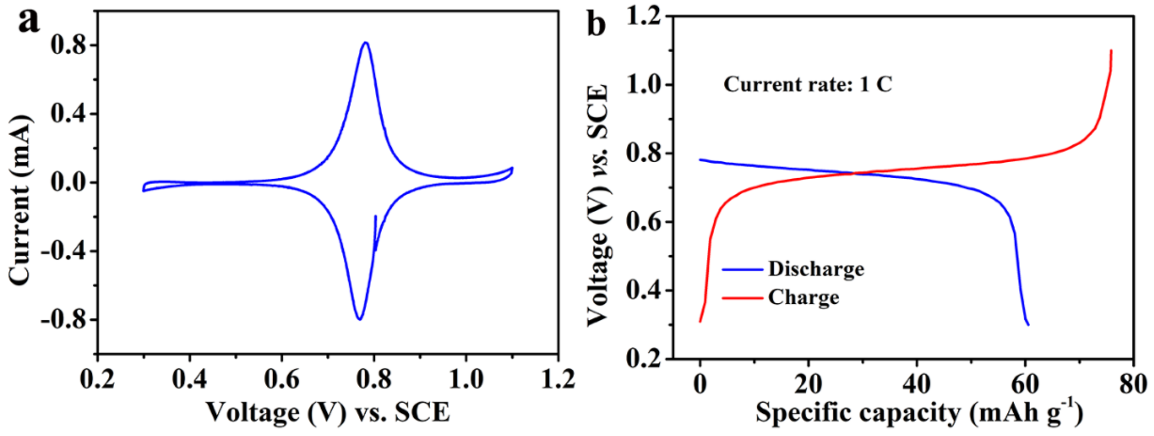

Fig. S6 CV and GCD curves of CuHCF during pretreatment process

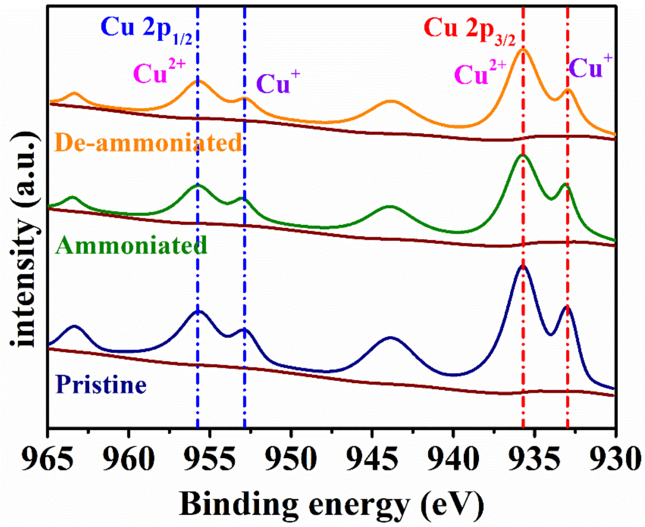

Fig. S7 Ex-situ XPS spectra for Cu 2p at pristine and ammoniated/de-ammoniated states

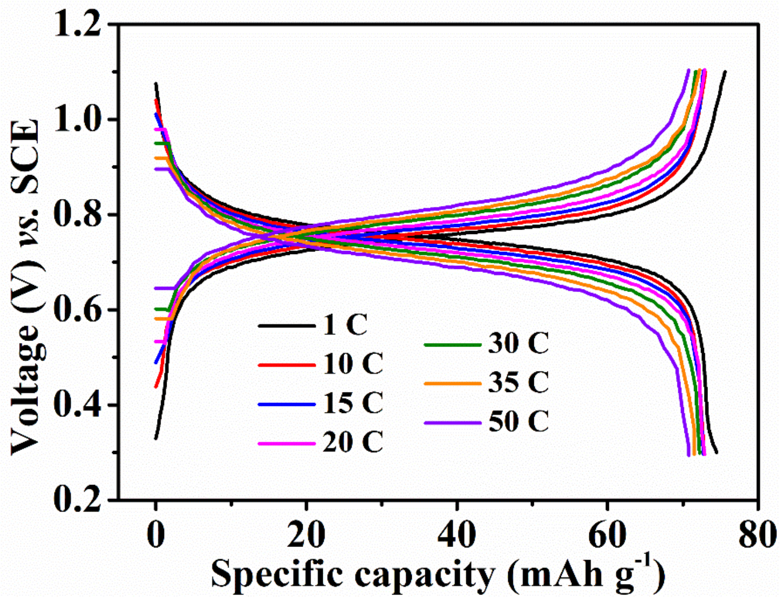

Fig. S8 Charge/discharge curves of CuHCF at current rates of 1, 10, 15, 20, 30, 35, and 50 C

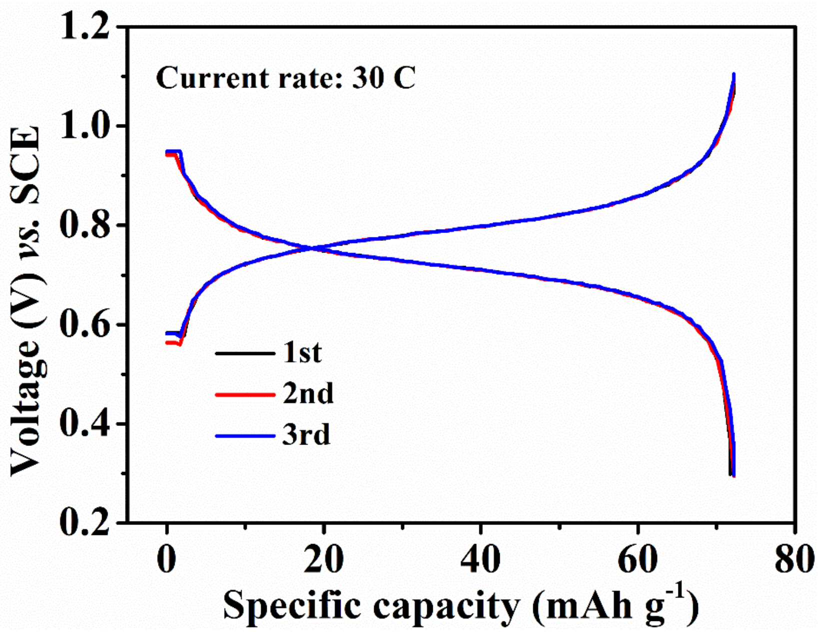

Fig. S9 The first three charge/discharge curves of CuHCF at current rate 30 C

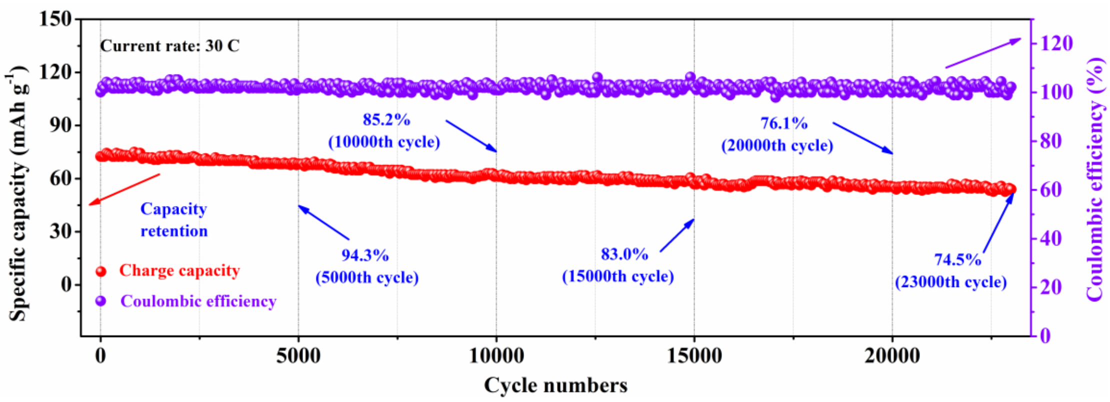

Fig. S10 Long-term cycling performance of CuHCF at high current rate of 30 C

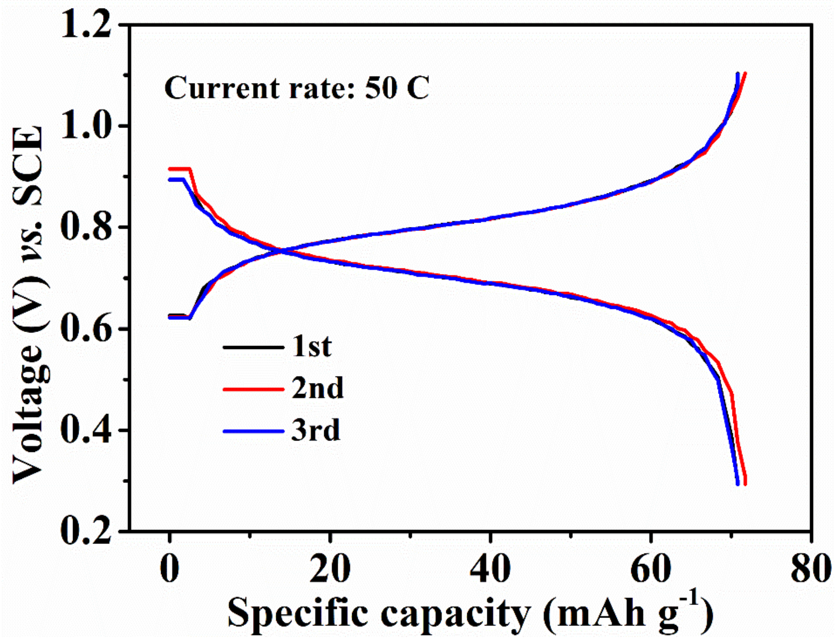

Fig. S11 The first three charge/discharge curves of CuHCF at current rate 50 C

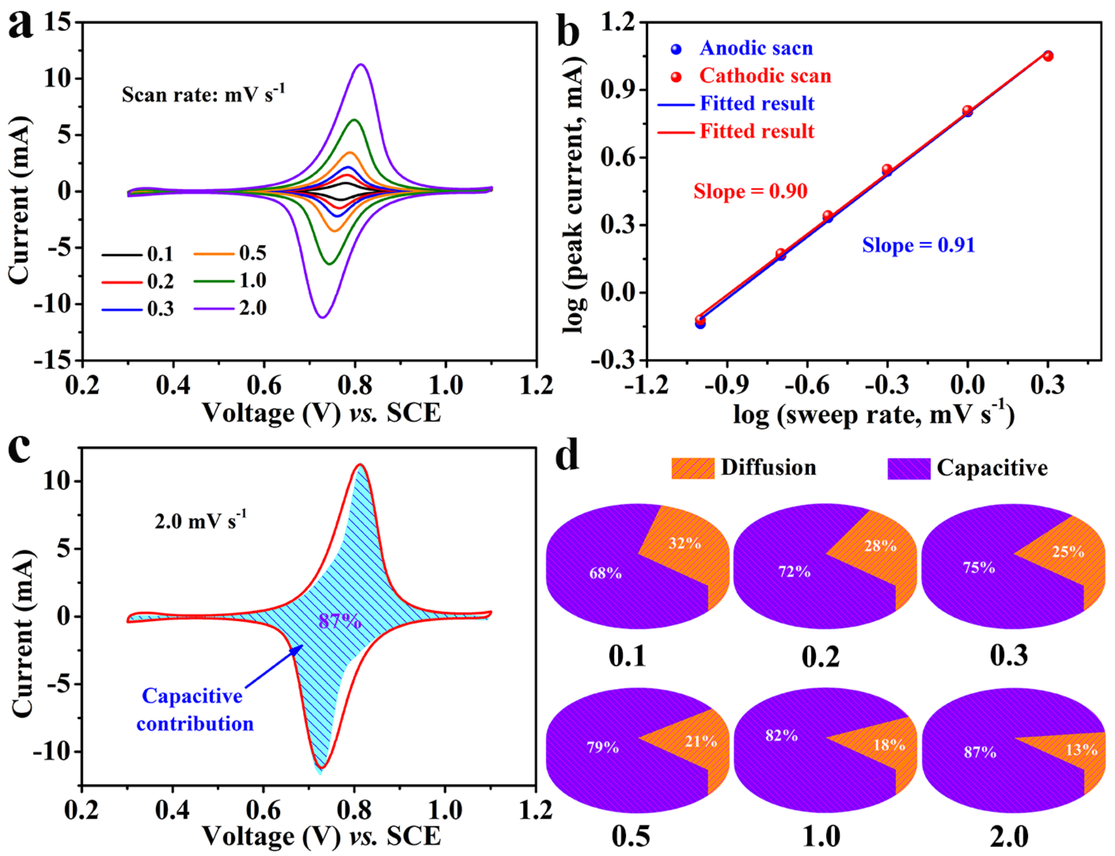

Fig. S12 Kinetics and quantitative analysis of NH_4^+ storage mechanism. **a** CV curves from 0.1 to 2.0 mV s^{-1}. **b** The relationship between logarithm cathodic peak current and logarithm scan rates. **c** Capacitive (shaded region) and diffusion-controlled contributions to charge storage of NH_4^+ at 2.0 mV s^{-1}. **d** Normalized contribution ratio of capacitive and diffusion-controlled capacities at different scan rates

With the increase of scan rates, the CV curves maintain the same shape. And the composition of charge storage can be roughly determined by measuring CV curves at different scan rates. In addition, for a given scan rate (υ), the relationship between peak current (i_p) and scan rate (υ) can be came down to the follow equations [S6],

$$i_p(\upsilon) = a\upsilon^b \qquad \text{(S1)}$$

$$\log(i_p) = \log(a) + b\log(\upsilon) \qquad \text{(S2)}$$

Where the b value is the slope of fitting line for $\log(i_p)$ *vs.* $\log(\upsilon)$ in equation (2). Besides, the diffusion and capacitive controlled progresses can be determined when the b values are 0.5 and 1.0, respectively [S7, S8]. The b values of 0.91 and 0.90 for the anodic and cathodic scans demonstrate the high capacitive controlled progresses during the ammnoiation/de-ammnoiation in CuHCF. And the capacitive contribution is further quantified according to the follow equations [S9, S10],

$$i_p(V) = k_1\upsilon + k_2\upsilon^{0.5} \qquad \text{(S3)}$$

$$i_p(V)/\upsilon^{0.5} = k_1\upsilon^{0.5} + k_2 \qquad \text{(S4)}$$

Where $k_1\upsilon$ and $k_2\upsilon^{0.5}$ represent the capacitive and diffusion contributions, respectively. The k_1 value can be obtained at each particular voltages by fitting $i_p(V)/\upsilon^{0.5}$ and $\upsilon^{0.5}$, thus determining the capacitive contribution at given scan rates.

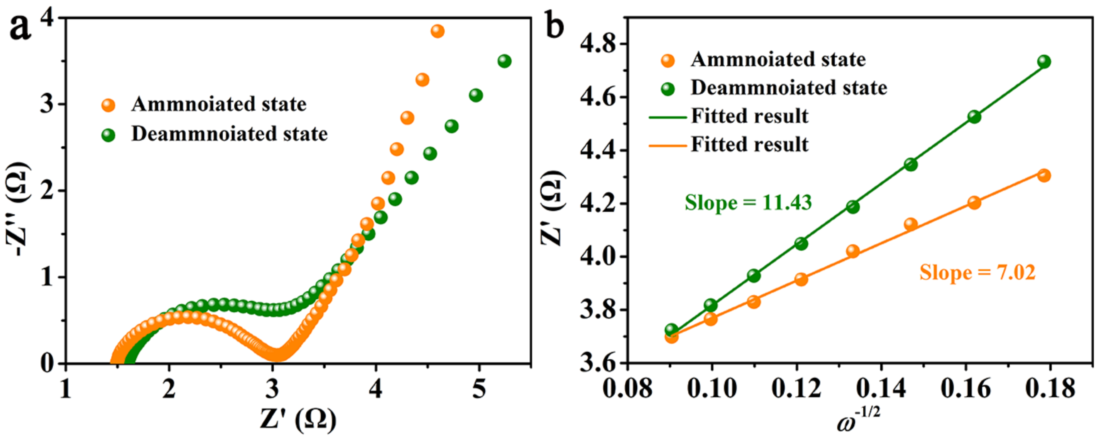

Fig. S13 a EIS patterns of CuHCF at ammnoiated and deammnoiated states. **b** The relationship between Z′ and $\omega^{-1/2}$ at low frequency

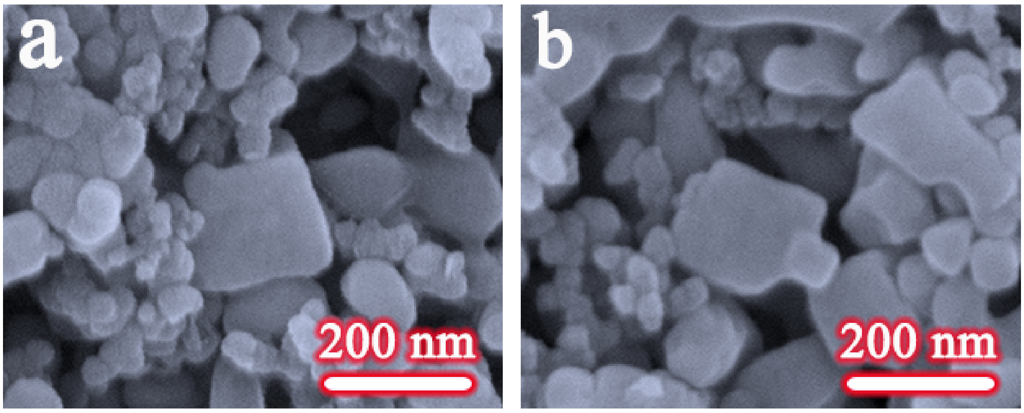

Fig. S14 a SEM image of pristine CuHCF electrode. **b** SEM image of CuHCF electrode after 3000 cycles

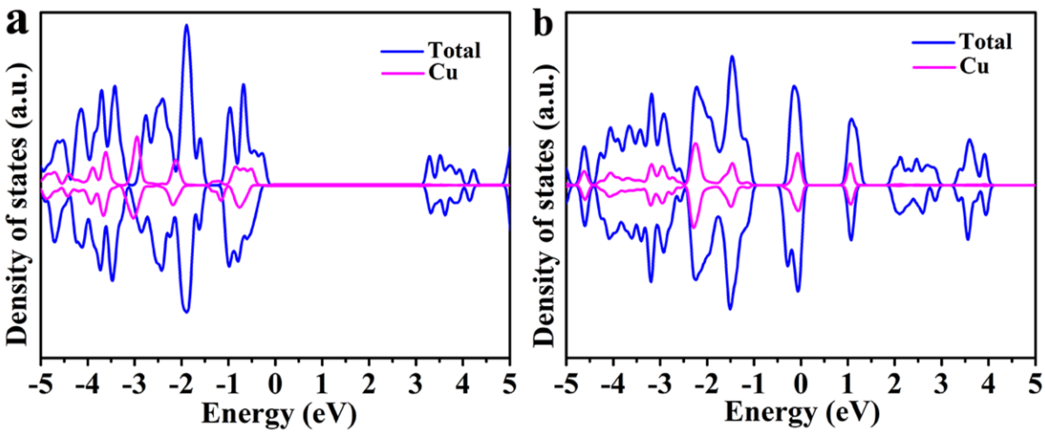

Fig. S15 Density of states for Cu atom in pristine and ammoniated CuHCF

Supplementary References

[S1] G. Kresse, J. Furthmuller, Efficiency of ab-initio total energy calculations for metals and semiconductors using a plane-wave basis set. Comp. Mat. Sci. **6**, 15 (1996). https://doi.org/10.1016/0927-0256(96)00008-0

[S2] G. Kresse, J. Furthmuller, Efficient iterative schemes for ab-initio total-energy calculations using a plane-wave basis set. Phys. Rev. B **54**, 11169 (1996). https://doi.org/10.1103/PhysRevB.54.11169

[S3] J.P. Perdew, K. Burke, M. Ernzerhof, Generalized gradient approximation made simple. Phys. Rev. Lett. **77**, 3865 (1996). https://doi.org/10.1103/PhysRevLett.77.3865

[S4] G. Kresse, D. Joubert, From ultrasoft pseudopotentials to the projector augmented-wave method. Phys. Rev. B **59**, 1758 (1999). https://doi.org/10.1103/PhysRevB.59.1758

[S5] P.E. Blöchl, Projector augmented-wave method. Phys. Rev. B **50**, 17953 (1994). https://doi.org/10.1103/physrevb.50.17953

[S6] R.T. Zheng, S.S. Qian, X. Cheng, H.X. Yu, N. Peng et al., $FeNb_{11}O_{29}$ nanotubes: Superior electrochemical energy storage performance and operating mechanism. Nano Energy **58**, 399 (2019). https://doi.org/10.1016/j.nanoen.2019.01.065

[S7] J. Wang, J. Polleux, J. Lim, B. Dunn, Pseudocapacitive contributions to electrochemical energy storage in TiO_2 (anatase) nanoparticles. J. Phys. Chem. C **111**, 14925 (2007). https://doi.org/10.1021/jp074464w

[S8] H. Kim, J. Hong, Y.-U. Park, J. Kim, I. Hwang et al., Sodium storage behavior in natural graphite using ether-based electrolyte systems. Adv. Funct. Mater. **25**, 534 (2015). https://doi.org/10.1002/adfm.201402984

[S9] M. Sathiya, A.S. Prakash, K. Ramesha, J-M. Tarascon, A.K. Shukla, V_2O_5-anchored carbon nanotubes for enhanced electrochemical energy storage. J. Am. Chem. Soc. **133**, 16291 (2011). https://doi.org/10.1021/ja207285b

[S10] Y.Q. Jiang, J.P. Liu, Definitions of pseudocapacitive materials: A brief review. Energy Environ. Mater. **2**, 30 (2019). https://doi.org/10.1002/eem2.12028
